# An enzyme in the kynurenine pathway that governs vulnerability to suicidal behavior by regulating excitotoxicity and neuroinflammation

**DOI:** 10.1038/tp.2016.133

**Published:** 2016-08-02

**Authors:** L Brundin, C M Sellgren, C K Lim, J Grit, E Pålsson, M Landén, M Samuelsson, K Lundgren, P Brundin, D Fuchs, T T Postolache, L Traskman-Bendz, G J Guillemin, S Erhardt

**Affiliations:** 1 Center for Neurodegenerative Science, Van Andel Research Institute, Grand Rapids, MI, USA; 2 Department of Physiology and Pharmacology, Karolinska Institute, Stockholm, Sweden; 3 Stanley Center for Psychiatric Research, Broad Institute of MIT and Harvard, Cambridge, MA, USA; 4 Psychiatric and Neurodevelopmental Genetics Unit, Center for Human Genetics Research, Massachusetts General Hospital, Boston, MA, USA; 5 Faculty of Medicine and Health Sciences Macquarie University, Sydney, NSW, Australia; 6 Institute of Neuroscience and Physiology, The Sahlgrenska Academy at Gothenburg University, Gothenburg, Sweden; 7 Department of Clinical and Experimental Medicine, Linköping University, Linköping, Sweden; 8 Division of Biological Chemistry, Innsbruck Medical University, Center for Chemistry and Biomedicine, Innsbruck, Austria; 9 Department of Psychiatry, University of Maryland School of Medicine, Baltimore, MD, USA; 10 Rocky Mountain MIRECC, Denver, CO, USA; 11 Section for Psychiatry, Department of Clinical Sciences, Lund University, Lund, Sweden; 12 NHMRC Centre of Research Excellence in Suicide Prevention (CRESP), Randwick, NSW, Australia

## Abstract

Emerging evidence suggests that inflammation has a key role in depression and suicidal behavior. The kynurenine pathway is involved in neuroinflammation and regulates glutamate neurotransmission. In the cerebrospinal fluid (CSF) of suicidal patients, levels of inflammatory cytokines and the kynurenine metabolite quinolinic acid (QUIN), an *N*-methyl-d-aspartate receptor agonist, are increased. The enzyme amino-β-carboxymuconate-semialdehyde-decarboxylase (ACMSD) limits QUIN formation by competitive production of the neuroprotective metabolite picolinic acid (PIC). Therefore, decreased ACMSD activity can lead to excess QUIN. We tested the hypothesis that deficient ACMSD activity underlies suicidal behavior. We measured PIC and QUIN in CSF and plasma samples from 137 patients exhibiting suicidal behavior and 71 healthy controls. We used DSM-IV and the Montgomery-Åsberg Depression Rating Scale and Suicide Assessment Scale to assess behavioral changes. Finally, we genotyped ACMSD tag single-nucleotide polymorphisms (SNPs) in 77 of the patients and 150 population-based controls. Suicide attempters had reduced PIC and a decreased PIC/QUIN ratio in both CSF (*P*<0.001) and blood (*P*=0.001 and *P*<0.01, respectively). The reductions of PIC in CSF were sustained over 2 years after the suicide attempt based on repeated measures. The minor C allele of the ACMSD SNP rs2121337 was more prevalent in suicide attempters and associated with increased CSF QUIN. Taken together, our data suggest that increased QUIN levels may result from reduced activity of ACMSD in suicidal subjects. We conclude that measures of kynurenine metabolites can be explored as biomarkers of suicide risk, and that ACMSD is a potential therapeutic target in suicidal behavior.

## Introduction

Suicide is defined as the intentional termination of one's own life, and constitutes the 10th leading cause of death globally.^1^ According to a recent report by the World Health Organization, >800 000 deaths by suicide occur around the world each year.^2^ Over the past decade, accumulating clinical data indicate that inflammation contributes to the pathophysiology of suicide. Recent meta-analyses on inflammation in suicidal patients conclude that there are aberrant cytokine levels in blood, cerebrospinal fluid (CSF), and post-mortem brain samples from suicidal patients.^3, 4^ Increased blood levels of tumor necrosis factor-α, interleukin-1β (IL-1β) and IL-6 are associated with suicidal ideation and behavior, and therefore these cytokines have been proposed to constitute biomarkers to help distinguish suicidal from nonsuicidal patients.^5, 6^ We have found that the CSF levels of IL-6 were increased threefold in patients who recently attempted suicide compared with healthy controls.^7^

**Figure 1:**
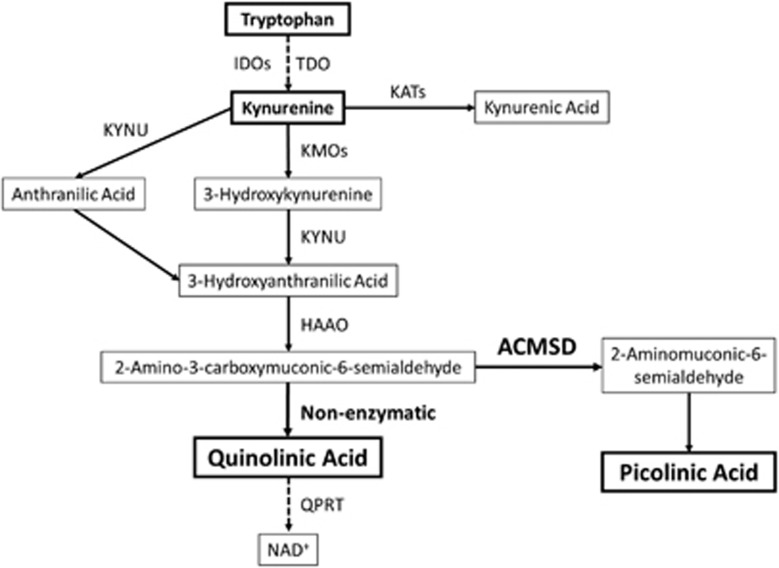
A simplified diagram of the kynurenine pathway. ACMSD, amino-β-carboxymuconate-semialdehyde-decarboxylase; HAAO, hydroxyanthranilate 3,4-dioxygenase; IDO, indoleamine 2,3-dioxygenases; KAT, kynurenine aminotransferases; KMO, kynurenine 3-monooxygenases; KYNU, kynureninase; NAD, nicotinamide adenine dinucleotide; QPRT, quinolinate phosphoribosyltransferase; TDO, tryptophan 2,3-dioxygenase.

Some of the effects of inflammation on mood and behavior may be mediated by kynurenine pathway metabolites, which modulate neuroinflammation and glutamate neurotransmission. More than 90% of dietary tryptophan is degraded along the enzymatic kynurenine pathway, and in the process several neuroactive compounds are formed, including quinolinic acid (QUIN) and picolinic acid (PIC)^8^ (Figure 1). Proinflammatory cytokines, particularly interferon-γ (IFN-γ), and also IL-1β and IL-6 activate the kynurenine pathway by inducing several enzymes, for example, indoleamine 2,3 dioxygenase (IDO) and tryptophan 2,3-dioxygenase (TDO).^9, 10^ QUIN is an *N*-methyl-d-aspartic acid receptor (NMDA-R) agonist that in the central nervous system is formed to a large extent within microglial cells. QUIN is a potent excitotoxin with proinflammatory and immunoregulatory properties.^9^ In addition to being a direct NMDA-R agonist, QUIN increases neuronal glutamate release and decreases glutamate uptake and recycling by astrocytes.^9^ Consequently, QUIN has dual effects on NMDA-R, both acting as an agonist and increasing extracellular glutamate levels. Ketamine, an NMDA-R antagonist, has proven effective in counteracting suicidal ideation in several clinical studies, highlighting the role of glutamate neurotransmission in suicidal patients.^11, 12^ In mice, inflammation-induced depressive-like behavior can be reversed by ketamine, providing further support for an association between inflammation, NMDA-R activation and depression.^13^ It has been proposed that production of the NMDA-R agonist QUIN is the mechanistic link between inflammation and symptoms of depression and suicidality.^14, 15^

We have discovered a threefold increase in the levels of QUIN in the CSF of suicidal patients compared with healthy controls.^15^ Other groups have also detected increased QUIN staining in post-mortem brain sections from patients with severe depression who committed suicide.^16^ QUIN is produced by the spontaneous conversion of the precursor metabolite 2-amino-3-carboxymuconate-6-semialdehyde (ACMS; Figure 1). However, ACMS can also be enzymatically processed by amino-β-carboxymuconate-semialdehyde-decarboxylase (ACMSD) to form PIC.^17^ In contrast to QUIN, PIC has been shown to possess neuroprotective properties in a number of studies, and is known to antagonize the effects of QUIN.^18, 19, 20^ ACMSD, expressed in liver, kidney and brain, has previously been predicted to have a key role in neuropsychiatric disorders because of its crucial position as a gatekeeper in the metabolism of tryptophan degradation along the kynurenine pathway.^21^ Still this enzyme has only recently begun to be investigated in neuropsychiatric disorders, and its functional effects have not yet been characterized. In the present study, we sought to characterize the biological underpinnings of the increased production of QUIN in patients with suicidal behavior. We hypothesized that QUIN is increased in both blood and CSF of suicidal patients due to decreased ACMSD activity, as reflected by reduced levels of the metabolite PIC and a reduced ratio of PIC/QUIN. We therefore quantified PIC levels in several cohorts of suicidal patients and healthy controls, both in direct conjunction to a suicide attempt and longitudinally for up to 2 years after the attempt. In addition, we examined the levels of tryptophan/kynurenine in these subjects as a measure of initial activation of the kynurenine pathway. We assessed the proinflammatory status in the blood by monitoring neopterin levels, a peptide synthesized by IFN-γ-stimulated macrophages.^22^ Finally, we examined the effect of *ACMSD* haplotypes, using representative single-nucleotide polymorphisms (tag SNPs), on suicidal behavior and levels of QUIN in CSF.

## Materials and methods

### Ethical approval and design of the study

Our study was carried out in accordance with ‘The code of ethics of the world medical association (Declaration of Helsinki)' for experiments including humans. The Regional Ethical Review Boards in Lund, Linköping, Malmö and Stockholm, approved the study. After complete description of the study, written informed consent was obtained from all subjects. Supplementary Figure 1 shows the different cohorts and the types of samples used in this study.

### CSF cohort

Sixty-four patients (30 men and 34 women) were enrolled following admission to Lund University Hospital after a suicide attempt (see below for definition) between 1988 and 2001. Psychiatric diagnoses and demographics of the subjects are displayed in Table 1. The patients underwent a washout period when they did not receive any antipsychotic or antidepressive medication (14.6±9 days, mean±s.d.). At the end of the washout period, lumbar punctures and psychiatric evaluations were carried out as below. Thirty-six healthy controls (29 men and 7 women) were recruited via the Psychiatric Clinics at the University Hospitals in Lund and Linkoping, Sweden, between 2003 and 2009. The controls did not suffer from any previous or ongoing psychiatric condition or substance abuse and were somatically healthy. They were thoroughly checked for psychiatric morbidity using the Structured Clinical Interviews for DSM Disorders (SCID I and II). All controls were free of medication. The levels of QUIN and cytokines were described before in this cohort.^7, 15^

### Plasma cohort

Seventy-three patients (31 men and 42 women) admitted to Lund University Hospital, Sweden, after a suicide attempt between 2006 and 2010 were enrolled. Only patients with an explicit intent were enrolled (see below, ‘Definition of suicide attempts'). The patients had various psychiatric diagnoses and the majority were treated with pharmacological drugs. Table 2 shows the demographics of the patients, including psychiatric and somatic diagnoses. Thirty-five healthy control subjects (16 men and 19 women) were randomly selected from the municipal population register in Lund, Sweden between 2008 and 2010. Exclusion criteria for the controls were the following: previous or ongoing psychiatric or somatic disorder; prior suicide attempts; prior psychiatric treatment; including psychotherapy, ongoing somatic illness or treatment (including painkiller or antibiotics), pregnancy, suicide or non-fatal suicidal self-directed violence in first-degree relatives, and sporadic recent drug use. Subjects who did not meet any exclusion criteria at the phone interview were further assessed for psychiatric and somatic pathology during an appointment with a research nurse and a resident or specialist in psychiatry. They were checked for alcohol abuse using the Alcohol Use Disorders Identification Test (AUDIT)^23^ with participants scoring 8 or above being excluded. A somatic examination was performed as described below for all subjects.

### Genotyping cohort

Whole blood for genotyping was available from 227 individuals, including 77 suicide attempters from both the CSF and the plasma cohort. In addition, a cohort of 150 population-based healthy controls was genotyped. These were randomly selected by Statistics Sweden (SCB) and contacted by mail with a request for them to contact study nurse if interested in participating in the study. Following a preliminary telephone screening, eligible persons were interviewed by experienced psychiatrists using Mini International Neuropsychiatric Interview to exclude psychiatric disorders. AUDIT and the Drug Use Disorders Identification Test (DUDIT) were used to exclude substance abuse. Apart from psychiatric disorders, other excluded diagnoses were neurological conditions other than mild migraines, untreated endocrinological disorders, autoimmune disorders, pregnancy and a family history of schizophrenia or bipolar disorder in first-degree relatives. All controls were born in Sweden, as were their parents (see also Jackobsson *et al.*^24^).

### Long-term CSF study

All patients were initially enrolled in the study on admission to Lund University Hospital after a suicide attempt and were subsequently followed for a maximum of 56 months after the attempt (mean 16 months). Twenty-nine patients (9 men and 20 women) with a mean age of 40±9 years (mean±s.d.) were included between 1987 and 1992. A total of 143 CSF samples were collected from these individuals at time points when they were also assessed using the Montgomery Asberg Depression Rating Scale (MADRS) and the Suicide Assessment Scale (SUAS) as described.^24^ The levels of inflammatory cytokines as well as QUIN and KYNA in the CSF of these patients have been previously reported.^25^

### Psychiatric assessment of patients

Briefly, after the suicide attempt, a psychiatrist diagnosed all patients according to the Diagnostic and Statistical Manual of Mental Disorders (DSM)-IIIR Axis I and II Disorders. The diagnoses were set by the psychiatrist after an ~2-h-long structured interview using the Comprehensive Psychiatric Rating Scale (CPRS) and the Structured Clinical Interview for DSM Disorders (SCID I and II). The CPRS is a widely used rating instrument in psychiatric research and consists of 65 scaled items, covering a wide range of psychiatric symptoms.^26^ We evaluated the severity of depressive symptoms using the Montgomery-Åsberg Depression Rating Scale (MADRS), which is a 10-item scale with a maximum score of 60 derived from CPRS.^27^ The Suicide Assessment Scale (SUAS) is an interview-based scale, consisting of 20 items assessing signs and symptoms related to current degree of suicidality and has a maximum score of 80.^28^ SUAS has been validated in predicting future suicides.^29, 30^

### Definition of suicide attempts

A suicide attempt was defined as ‘situations in which a person has performed an actually or seemingly life-threatening behavior with the intent of jeopardizing his/her life'.^31^ Moreover, to be enrolled, the intent to commit suicide had to be explicit upon the clinical interview. Patients who did not state a clear intent were not enrolled. Suicide attempts were classified into violent and non-violent acts as previously defined.^32^ Drug-overdoses, single wrist-cuts or a combination are considered non-violent suicide attempts, whereas all other methods (for example, hanging, drowning and gas poisoning) were classified as violent.

### Somatic examination

All patients and controls in the plasma and CSF cohorts underwent a general physical examination. In order to identify subjects with potential infections at the time of the lumbar punctures and blood samples, we analyzed blood samples for white blood cell count, erythrocyte sedimentation rate or C-reactive protein, and the subjects were checked for fever. No evidence of ongoing clinical infection was found, as defined by the normal reference intervals of these parameters. A complete medical history was taken. Somatic diagnoses of the subjects are shown in Tables 1 and 2.

### CSF and blood sampling

CSF was obtained from the L4–L5 interspace, using a standardized protocol. The CSF samples were drawn between 0800 and 1100 hours after a night of fasting and bed rest. The CSF samples were kept on ice during the sampling and thereafter immediately aliquoted and frozen at −80 °C. Blood samples were collected between 0730 and 0800 hours after a night of fasting and bed rest. The suicidal patients were psychiatric inpatients, whereas the control-groups had spent the night at home. The blood was placed on ice and centrifuged (3000 r.p.m., at +4 °C) within 1 h. Plasma was collected and stored at −80°C within 1 h after sampling. All blood and CSF samples were kept at −80 °C until analyses. The analysis of PIC, QUIN, kynurenine and tryptophan levels in blood and CSF as well as genotyping were performed by staff blinded to the experimental groups, with samples coded.

### Biological assays

#### Gas chromatography–mass spectrometry

To extract KP metabolites, samples were treated with trichloroacetic acid at a final concentration of 5% (*w+v*) in equal volume and incubated in ice for 5 min, vortexed and then centrifuged (4 °C) for 10 min at 12 000 r.p.m. Supernatant was carefully extracted and filtered with a 0.22 μm syringe filter (Millipore-Merck, Billerica, MA, USA). QUIN and PIC levels in CSF and plasma were examined using gas chromatography–mass spectrometry with the spectrometer operating in electron-capture negative-ionization mode. The method used is described in Smythe *et al.*^33^ The internal standards were purchased from Le Research (St Paul, MN, USA). Trifluoroacetic anhydride and 1,1,1,3,3,3-hexafluoro-2-propanol of GC derivatization grade, QUIN, PIC and other organic solvents of analytical-grade were all obtained from Sigma-Aldrich (St Louis, MO, USA). One microliter of sample was injected into an Agilent 6890 gas chromatograph, interfaced to an Agilent 5973 mass selective detector via an auto-sampler Agilent Technologies 7683, and controlled using Agilent ChemStation software (Agilent, Santa Clara, CA, USA). The inter- and intra-assay coefficient of variation was <5%.

#### Ultrahigh-performance liquid chromatography

Kynurenine and tryptophan were concurrently measured using Agilent 1290 ultrahigh-performance liquid chromatography as previously described.^34^ Briefly, isocratic elution of kynurenine and tryptophan were achieved using a reverse-phase Poroshell RRHT C18, 1.8 μm 2.1x150 mm column (Agilent Technologies) maintained at 38 °C for 12 min run time at a flowrate of 0.75 ml min^−1^ using 0.2 mm sodium acetate (pH 4.65) as mobile phase. Kynurenine was detected using ultraviolet wavelength at 365 nm, whereas tryptophan was detected using fluorescence intensity set at excitation/emission wavelength of 280/438 nm. Mixed standards of both metabolites were used for a six-point calibration curve in order to interpolate the quantity of the sample readout using Agilent OpenLAB CDS Chemstation (Edition C.01.04) to analyze the chromatogram. The inter- and intra-assay coefficients of variation were within the acceptable range of 3–7%.

#### Enzyme-linked immunosorbent assays

Neopterin concentrations were determined by a commercially available ELISA 99R.096 (BRAHMS, Henningsdorf, Germany) following the manufacturers' instructions. Sensitivity of the test was 2 nmol l^−1^. All samples were within the detectable range.

### Genotyping

Tag SNPs for *ACMSD* (±20 kB) were chosen using Tagger (HapMap analysis panel: CEU, *r*^*2*^ threshold=0.8).^35^ SNP genotyping (rs4953936, rs1954874, rs2121337, 6722883, rs6730306, rs10928521, rs6714498 and rs10176573) were carried out using 5' nuclease assay chemistry with custom KASP technology probes^36^ at the Genomics Core Facility at the University of Gothenburg, Sweden. One SNP (rs6730306) failed at assay design. No systematic difference in individual missingness percentage was seen across the two cohorts of patients and controls. All markers had <10% missing data and none displayed a significant deviation from the Hardy–Weinberg equilibrium.

### Statistical analysis

All statistical analysis, except the longitudinal CSF analysis, was performed using the Statistical Package for the Social Sciences (IBM SPSS Statistics 20.0 (IBM SPSS, Chicago, IL, USA)). The longitudinal analysis was performed using R v 3.2.2 (https://www.r-project.org/). The power calculation was based on variance and mean levels of inflammatory factors in suicide attempters versus controls detected in our previous studies. Mean PIC case concentration was estimated at 175 nm and control concentration at 250 nm. Pooled sample s.d. across the two samples was estimated to be 150 nm. Power was computed for a two-tailed *t*-test and 84% power is achieved with *n*=35 per group. In plasma, PIC, QUIN and the kynurenine/tryptophan ratios displayed a non-normal distribution and non-parametrical tests were used for groupwise comparisons and correlations. For linear regression, the measures were transformed into normal distribution by logarithm. CSF analytes displayed a normal distribution. Groupwise comparisons were performed using independent samples *t*-test or *U*-tests. Correlations were performed using Pearson's R or Spearman's Rho. A linear mixed-effects model with random slopes was used to determine whether CSF levels changed over the 2 years following a suicide attempt. The model was originally adjusted for age and gender; although as no evidence was found that these impacted CSF levels, they were subsequently omitted in the final model. The delta method was used to test whether controls differed from the index CSF levels of suicide attempters, where the mean index CSF was estimated by the linear mixed-effects model. For the genetic analyses, we used the subsample of patients and controls with available CSF QUIN, PIC and kynurenine data. We applied simple linear regression models to study the effect of *ACMSD* tag SNPs variants on CSF levels of QUIN, the QUIN/PIC ratio (log transformed), and kynurenine. Common genetic variants that were significantly associated with CSF levels of QUIN, after adjustment for multiple comparisons (Bonferroni correction), were analyzed in cases versus controls using logistic regression with adjustment for age and sex. All genetic analyses were performed using an additive model. All reported *P*-values are two-sided and analyses were performed using either IBM SPSS Statistics 20.0 or PLINK (http://pngu.mgh.harvard.edu/purcell/plink/).^37^

## Results

### Potential confounders

The levels of PIC in CSF were associated with the age of the subject (Pearson's R −0.25, *P*<0.05), but not with gender, body mass index or age of samples (Student's *t*-test and Pearson's R, not significant (NS)). The PIC data in CSF was therefore corrected for subject age in all subsequent groupwise comparisons. The kynurenine/tryptophan ratio in CSF was associated with age and gender of subjects and was therefore corrected for these covariates in groupwise comparisons. PIC in plasma was not associated with any of the mentioned co-variates, whereas the plasma kynurenine/tryptophan ratio and plasma QUIN were associated with the age of subjects and therefore corrected for this in all subsequent groupwise comparisons.

### CSF cohort

**Figure 2:**
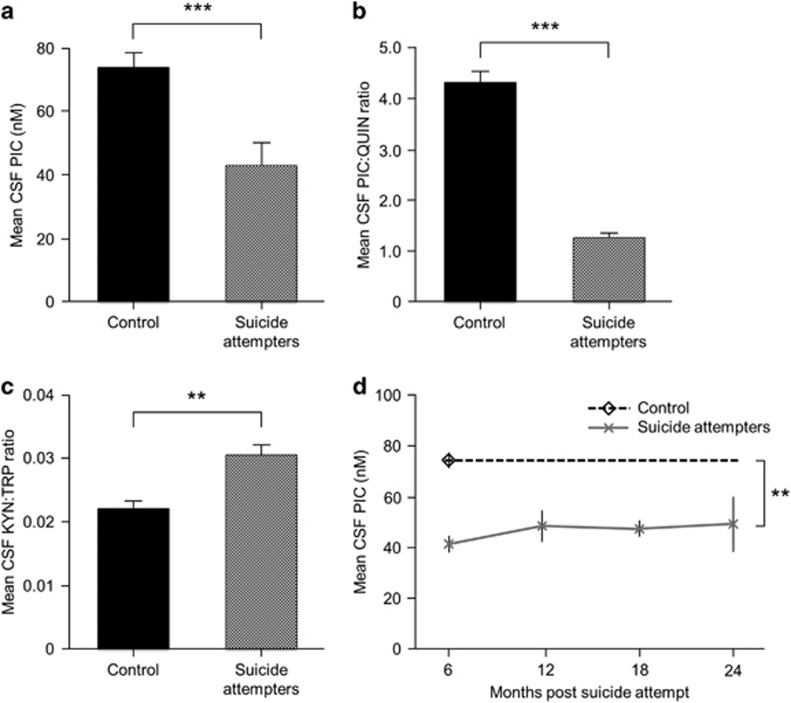
Kynurenine metabolites in the cerebrospinal fluid (CSF) of suicide attempters and healthy controls. (**a**) CSF picolinic acid (PIC). (**b**) CSF picolinic acid/quinolinic (PIC/QUIN) acid ratio. (**c**) CSF kynurenine/tryptophan ratio (KYN/TRP). (**d**) CSF PIC in suicide attempters over time compared with a single time point measured in control subjects. PIC was lower in suicide attempters than in healthy controls and did not fluctuate over time. **(a**–**d**) **P<0.01; ***P<0.001 suicide attempters vs controls. All values represent mean (+s.e.m.).

We found that the levels of PIC were significantly decreased in the CSF of suicide attempters (*n*=64) compared with healthy controls (*n*=36*; t*-test, *P*<0.001, *n*=100; Figure 2a). We recently reported that CSF QUIN is increased in the same patients.^15^ The PIC/QUIN ratio was consequently reduced in the suicidal patients, indicative of impaired activity or reduced biological levels of the enzyme ACMSD (*T*-test, *P*<0.001; Figure 2b).

The CSF kynurenine/tryptophan ratio was increased in suicide attempters compared with healthy controls (*t*-test, *P*<0.01, *n*=94; Figure 2c), indicating an activation of the kynurenine pathway.

No significant differences in CSF PIC levels were found between the five largest diagnostic groups of the patients (major depressive disorder, depression not otherwise specified, adjustment disorder, personality disorder and substance abuse; one-way analysis of variance (ANOVA), NS). The mean CSF PIC concentrations in the main diagnostic groups and controls are shown in Table 1. There were no correlations between the levels of PIC in CSF and the severity of psychiatric symptom load as assessed with total scores of CPRS, SUAS or MADRS (*n*=53 for SUAS, 55 for MADRS and CPRS, Pearson's R, NS).

There was no difference in CSF PIC levels between violent (*n*=16) and non-violent attempts (*n*=48), or between patients who had previously performed attempts (*n*=22) versus first-time suicide attempters (*n*=42) (*t*-tests, NS).

The patients in the CSF cohort had gone through a washout period of psychotropic medications including anti-depressants and neuroleptics, although they were allowed to use tranquilizers. Twenty-six of the patients used benzodiazepines intermittently either for anxiety or sleeping aid. There was no difference in CSF PIC levels between patients who did use benzodiazepines versus patients who did not (*n*=38, *t*-test, NS). A total of nine patients took other medications (Table 1). The statistical analyses were repeated without these nine patients, which did not alter the significant results.

### Longitudinal CSF follow-up study

A total of 29 patients contributed to a follow-up study, giving CSF samples at several random occasions over a 2-year period following a suicide attempt. Divided into four time periods (up to 6, 12, 18 and 24 months post suicide-attempt, respectively), the mean CSF PIC values of the patients were significantly lower than the value obtained from healthy controls (one-way ANOVA *P*<0.0001; all four individual time periods *P*<0.01, Bonferroni *post hoc* correction; Figure 2d).

As a secondary analysis of the same population, a linear mixed-effects model with random slopes was fit to determine whether PIC CSF scores changed over time for the patient group, taking all samples into account, at each individual time point. PIC levels did not change significantly over time (estimate=0.56 nm per month, s.e.m.=0.34, *P*=0.11) (Supplementary Figure 2). The delta method determined that patient index scores differed from the control group by an average of 34.82 nm (*P*=0.0039, s.e.m.=12.08). There was no evidence of a gender difference in either the control group or the patient group.

### Plasma cohort

**Figure 3:**
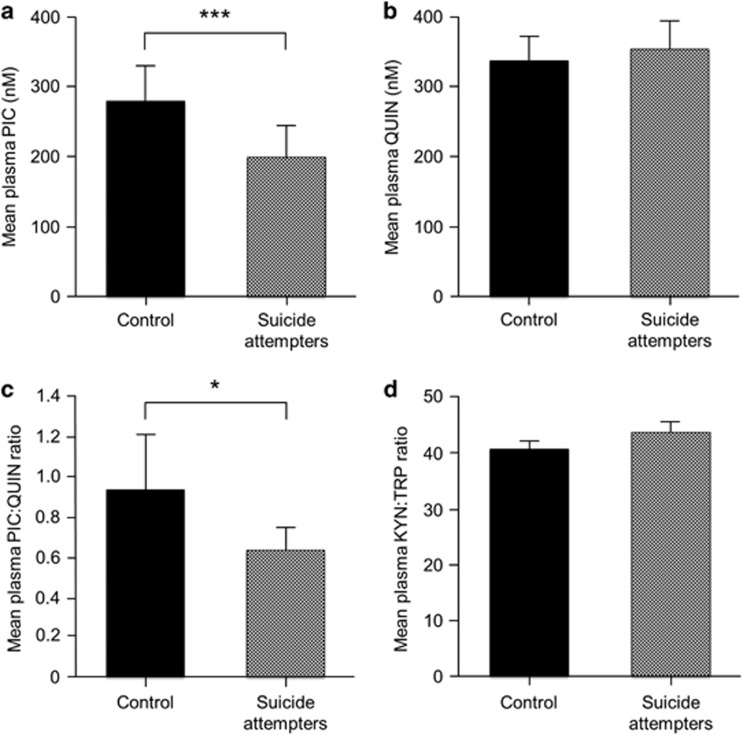
Kynurenine metabolites in the plasma of suicide attempters and healthy controls. (**a**) Plasma picolinic acid (PIC). (**b**) Plasma quinolinic acid (QUIN). (**c**) Plasma PIC/QUIN ratio. (**d**) Plasma kynurenine/tryptophan ratio. (**a**–**d**) *P<0.05; ***P<0.001 suicide attempters versus controls. All values represent mean (+s.e.m.).

Similar to the results in the CSF cohort, the levels of PIC were decreased in plasma of suicide attempters (*n*=73) compared with controls (*n*=35; *U*-test, *P*=0.001; Figure 3a). There was no difference in the levels of plasma QUIN (NS; Figure 3b). The PIC/QUIN ratio was reduced in the plasma of the suicide attempters compared with the healthy controls (*U*-test, *P*<0.05; Figure 3c). There were no differences in the degree of IDO/TDO activation in the peripheral blood between the suicide attempters and the healthy controls as indicated by an unaltered kynurenine/tryptophan ratio in plasma (*U*-test, NS, Figure 3d). However, we observed that the neopterin levels in plasma were positively associated with both the kynurenine/tryptophan ratio (Spearman's Rho=0.63, *P*<0.0005) and plasma QUIN (Spearman's Rho=0.39, *P*<0.01) in the patients (*n*=54) but not in the healthy controls (*n*=29).

Table 2 shows the PIC and QUIN levels of the patients and controls in the plasma cohort, separated by psychiatric and somatic diagnoses. As for the CSF cohort, there were no significant differences among the main diagnostic groups of patients (major depressive disorder, adjustment disorder, substance abuse and bipolar disorder; depressive episode) (one-way ANOVA, NS). Similarly, the QUIN levels in plasma did not differ depending on diagnostic subgroup (one-way ANOVA, NS). There was no correlation between the levels of PIC in plasma and CPRS, MADRS or SUAS scores in the patient group (Spearman's R, NS). The patients in the plasma cohort were on different psychotrophic medications, indicated in Table 3. There was no effect of any of the groups of medications on the PIC levels in plasma except for propiomazine (linear regression, Table 3). Patients (*n*=22) that received propiomazine, an antihistamine used for improved sleep, had higher levels of PIC in plasma. Because this effect cannot be a confounder of the result of significantly reduced PIC in plasma of patients, we did not correct for this effect. A total of 14 patients were untreated (that is, received none of the medications listed in Table 3). There was no significant difference in the plasma levels of PIC or QUIN between these untreated patients and patients on psychotrophic medication (*n*=58; *U*-test, NS).

### Genetic variation in *ACMSD* and suicidal behavior

First we studied the effect of *ACMSD* variants on CSF QUIN levels in all genotyped patients and controls with available CSF data (*n*=34; 10 controls and 24 patients). Among the seven tag SNPs only rs2121337 (minor allele frequency=0.14) remained significant post Bonferroni correction (*β*=0.4; *P*=0.04) (unadjusted *P*-value = 0.006). Further, adding age and sex as covariates had little impact on the association (*β*=0.5; *P*=0.01). Using the same sample, the minor C allele of rs2121337 was also associated with an increased CSF QUIN/PIC ratio (*β*=0.4; *P*=0.02; Supplementary Table 1). In line with these findings, the C allele was more common among the 72 genotyped suicide attempters than in our control sample of 135 healthy individuals (OR=2.0; *P*=0.03).

## Discussion

To our knowledge, our study provides the first evidence of reduced levels of PIC in CSF and blood in conjunction to a suicide attempt in two independent cohorts, as well as over a longitudinal follow-up period in patients initially enrolled in the CSF cohort. Moreover, we observed the same biological alterations in suicide attempters that were medication free (the CSF cohort) and in attempters that received different psychotropic medications (the plasma cohort). Our pilot genotyping study indicated that a SNP of *ACMSD,* rs2121337, was coupled to increased QUIN in CSF and was more prevalent in suicide attempters than healthy controls. Hence, we obtained support for our primary hypothesis, namely that a reduced ACMSD activity underlies excess QUIN production observed in patients exhibiting suicidal behavior. The reduced activity could be due to either a decreased enzymatic function or to a reduced tissue expression of the enzyme. As QUIN is an NMDA-R agonist and has additional enhancing effects on glutamate neurotransmission and neuroinflammation, reduced ACMSD function could contribute to a vicious circle with increased neuroinflammation being established in vulnerable patients. Altogether, our data indicate that ACMSD is a potential regulator of vulnerability/resilience to suicidal behavior upon inflammatory challenges.

ACMSD is expressed in liver and kidney as well as in neurons and astrocytes of the central nervous system.^38^ The expression and activity may also be induced in other tissues based on dietary or metabolic conditions,^39, 40, 41, 42, 43^ although the full tissue range of expression still remains to be characterized. The biological factors regulating the expression and activity of ACMSD in neurons and astrocytes are not yet well established. Genetic variants of ACMSD have previously been associated with differential expression of the enzyme in brain tissue in patients with Parkinson's disease.^44^ The purpose of including the tag SNP data in our current study was to perform a small, hypothesis driven verification of a possible involvement of a few SNPs in the *ACMSD* region. Our analyses differ from a genome-wide association study approach as we only included *ACMSD* tag SNPs in our analyses and selected candidate risk variants based on association with QUIN levels in CSF. Our sample size using this approach is inevitably limited and our data have to be interpreted with caution until replicated in larger studies. Nonetheless, these genetic analyses complement our patient versus controls analyses of CSF and plasma concentrations, and provide further support for a direct role of ACMSD function in suicidal behavior.

We studied the levels of PIC in CSF in a follow-up study over 2 years after an initial suicide attempt. Because the patients were not actively suicidal at the follow-up time points, we were able to study the CSF levels of PIC without any potential influence of a physical suicide attempt (for example, by intoxication or direct injury). We observed that the levels of PIC in CSF were reduced compared with controls at all time points, which indicates that decreased ACMSD activity may be a trait-marker of patients prone to suicidal behavior, rather than a state-marker. One could propose that an innate reduced biological activity of the ACMSD enzyme would make patients more susceptible to inflammatory conditions, as they would produce more QUIN upon inflammatory stimuli. In fact, we have shown that in patients with elevated QUIN the depressive and suicidal symptoms fluctuated over time with varying degree of inflammation in the CSF and blood.^25^ In addition, as the increased production of QUIN is by itself proinflammatory it can lead to increased microglial activation and secretion of proinflammatory cytokines.^9^ Thus, a reduced amount or activity of ACMSD within the central nervous system can predispose to the development of a chronic inflammatory environment, potentially with the capability of becoming self-sustained even after cessation of proinflammatory stimuli originated in the periphery. Conversely, in healthy subjects, constitutionally high ACMSD activity could be protective, contributing to shunting of kynurenine metabolism toward the production of the neuroprotective PIC and away from the neurotoxic QUIN, preventing a vicious circle generating inflammation and inflammatory metabolites.

We have previously shown that the degree of symptoms in patients exhibiting suicidal behavior is related to inflammation and QUIN levels in CSF.^7, 15, 25^ Therefore, our results primarily suggest that ACMSD could be important in the pathogenesis of suicidality based on its capability to reduce QUIN levels. Indeed, a study by Walker *et al.*^13^ showed that depressive-like behaviors induced by endotoxin injections (inducing inflammation) in rodents were reversed by the effects of ketamine on the NMDA receptor. Ketamine is an NMDA-R antagonist, with the potential of counteracting effects of the NMDA-R agonist QUIN. However, one should also consider that PIC itself could influence the pathogenesis of psychiatric disease. The neuroprotective mechanisms of action of PIC are not fully understood, but it is well known that PIC acts as a metal chelator.^17^ Bound to chromium, PIC has been shown to reduce symptoms in patients with atypical depression.^45^ PIC also exerts growth-factor-like properties, by inducing the production of macrophage inflammatory protein-1α and -1β as well as vascular endothelial growth factor, a potent neuroprotective agent,^46, 47^ and can moreover induce the differentiation of stem cells.^48^ Rodent studies have indicated that PIC may protect against depressive- and anxiety-like behaviors.^49, 50, 51^

Although the findings of reduced PIC and increased QUIN are most pronounced in the CSF of the suicide attempters, the PIC levels are also significantly reduced in peripheral blood, opening up the possibility of assessing PIC, or the ratio of PIC/QUIN in the blood as a potential risk marker of suicidal behavior. Sublette *et al.*^52^ have previously demonstrated that the kynurenine pathway is induced in the peripheral blood of suicidal patients, as evidenced by an increased kynurenine/tryptophan ratio, although they did not attempt to measure QUIN or PIC. In the current study, we observed increased kynurenine/tryptophan ratio in the CSF of the patients, but not in their peripheral blood; however, we found that the peripheral ratio was significantly associated with plasma neopterin levels in suicide attempters. Neopterin is synthesized and released primarily from human monocyte-derived macrophages and dendritic cells upon activation with IFN-γ^22^ therefore, elevated neopterin levels are usually accompanied by enhanced tryptophan breakdown because indoleamine 2,3 dioxygenase is upregulated by the same proinflammatory stimuli.^53^ This finding demonstrates a proinflammatory status in the blood of suicide attempters, so although our primary indicative is central nervous system inflammation, our study also points towards involvement of the peripheral immune system in the suicide attempters. In line with these findings it is important to note that the decreased activity or expression of ACMSD is readily detectable in blood by using PIC and QUIN levels as biomarkers. Therefore, we have identified both a plausible biological mechanism for the increased QUIN levels detected in suicide attempters (reduced activity or expression of ACMSD) and a peripheral biomarker that might indicate suicide risk (the plasma PIC/QUIN ratio).

Even though this study uses multiple cohorts and analyses both longitudinal and cross-sectional data as well as genotyping, we do not test any direct causality of increasing or decreasing the activity of ACMSD. Such mechanistic studies of causality should be undertaken in animal models of depressive-like behavior, and would further strengthen the implication of ACMSD as a potential target for pharmacological modulation in patients with suicidal behavior. Moreover, we do not know whether exogenous inflammation was the initial trigger of the observed changes in the patients, or whether a decreased enzymatic activity of ACMSD is sufficient to trigger a proinflammatory environment by itself. As we did not include any psychiatric control group, it will be important to define whether the changes in ACMSD biological activity are specific for suicidal behavior, or whether they also extend to other underlying psychiatric conditions. Notably, we did not detect any differences in the levels of PIC in CSF or blood between any of the diagnostic groups assessed here, which may suggest that changes in the kynurenine pathway are specifically associated with suicidal behavior, not with the primary diagnosis. A similar observation was made by Steiner *et al.*,^54^ who found post-mortem signs of brain inflammation in patients who died from suicide, irrespective of their primary diagnosis.

In conclusion, we have found that the neuroinflammation and excess QUIN previously observed in patients exhibiting suicidal behavior may be due to deficient activity of ACMSD, an enzyme of the kynurenine pathway that regulates the formation of the NMDA-R agonist and neurotoxin QUIN. Modulating ACMSD might represent a future strategy for the development of novel anti-suicidal pharmacological tools. Blood measurements of PIC and QUIN might prove useful as biomarkers in order to identify patients vulnerable to develop suicidal behavior.

## Figures and Tables

**Table 1:** Diagnoses, demographics, mean CSF PIC and medication list of the CSF cohort

*Psychiatric diagnoses*	*PIC mean nM (s.e.m.)*	*Age-corrected PIC*	*Age (range)*	*Somatic diagnoses*	*Medication*
Major depressive disorder (*n*=22)	43.8 (−8.5)	−0.18	41 (9–71)	Gastritis (*n*=1) Chronic pain (*n*=1) Migraine (*n*=1)	Benzodiazepine (*n*=7) Tricyclic (*n*=2)
Dysthymia (*n*=4)	33.9 (–8.4)	−0.53	40 (23–72)	Gastritis (*n*=1)	Benzodiazepine (*n*=1)
Substance abuse (*n*=6)	39 (−7.6)	−1.8	50 (28–61)	Chronic headache (*n*=1) Diabetes (*n*=1)	Benzodiazepine (*n*=3) Propiomazine (*n*=2) Dextropropoxyphene (*n*=1)
Adjustment disorder (*n*=11)	33.4 (3.6)	−0.73	30 (19–49)	Arthralgia (*n*=1)	Benzodiazepine (*n*=3) Omeprazole (*n*=1) Ranitidine (*n*=1) Doxycycline (*n*=1)
Anxiety disorder (*n*=1)	19.1	−0.84	49	—	Benzodiazepine (*n*=1)
Psychotic disorder (*n*=3)	40.2 (7.7)	−0.49	31 (26–35)	Peptic ulcer (*n*=1)	Benzodiazepine (*n*=2) Thioridazine (*n*=1)
Depression NOS (*n*=9)	32.1 (5.3)	−0.77	30 (22–42)	Migraine (*n*=1) Gastritis (*n*=1)	Benzodiazepine (*n*=5)
Personality disorder (*n*=8)	68.6 (20.0)	0.51	35 (23–54)	—	Benzodiazepine (*n*=4)
Controls (*n*=36)	73.6 (1.9)		30 (18–66)	—	—

Abbreviation: CSF, cerebrospinal fluid; NOS, not otherwise specified; PIC, picolinic acid.

The levels of PIC in the CSF were associated with the age of the subject and so they were corrected for age in subsequent analysis. Patients underwent a washout period (14.6±9 days, mean±s.d.) when they did not receive any antipsychotic or antidepressive medications prior to the collection of CSF.

**Table 2:** Diagnoses, demographics, mean plasma PIC and QUIN and medication list of the plasma cohort

*Psychiatric diagnoses*	*PIC mean nM (s.e.m.)*	*QUIN mean nM (s.e.m.)*	*Age (range)*	*Somatic diagnoses*
Major depressive disorder (*n*=15)	183.9 (13.9)	339.8 (32.9)	43 (20–67)	Anemia (spherocytosis) Liver transplant
Dysthymia (*n*=3)	118.8 (26.0)	321.2 (83.5)	44 (26–59)	Hypothyreosis Migraine
Bipolar disorder (*n*=21)	185.9 (16.8)	355.3 (18.3)	37.5 (20–67)	Fibromyalgia Migraine (*n*=2) Hypothyreosis Arthrosis Psoriasis Allergy Ischemic heart disease
Substance abuse (*n*=8)	190.8 (22.1)	294.3 (29.5)	44.5 (18.61)	Diabetes Arthrosis
Adjustment disorder (*n*=7)	143.6 (20.5)	271.6 (25.6)	41 (23–61)	Allergy
Anxiety disorder (*n*=5)	438.5 (323.6)	434.7 (138.9)	38 (20–73)	Diabetes Hypothyreosis
Psychotic disorder (*n*=3)	230.2 (17.8)	324.7 (40.0)	37 (23–49)	Diabetes
Depression UNS (*n*=4)	178 (25.4)	363.1 (111.8)	25.5 (21–39)	—
Personality disorder (*n*=2)	214.1 (76.1)	904.7 (596.5)	35.5 (25–46)	Lactose intolerance
No main psychiatric diagnosis (*n*=5)	177.4 (22.4)	264.7 (26.7)	37 (24–57)	Allergy (*n*=3) Migraine (*n*=2) Asthma
Controls (*n*=29)	285.2 (31.5)	329.7 (18.9)	40 (19–66)	—

Abbreviation: PIC, picolinic acid; QUIN, quinolinic acid.

**Table 3:** Effect of medication class on PIC levels in plasma

*Medication class*	*Number of individuals*	*Standardized beta*	*Significance (*P*-value)*
SSRI	30	−0.14	0.25
SNRI	25	−0.02	0.86
Neuroleptic	13	0.11	0.37
Anti-epileptic	10	−0.06	0.62
Hydroxyzine	12	0.2	0.08
Propiomazine	22	0.36	0.003
Benzodiazepine	43	−0.04	0.75
Anti-inflammatory	12	0.05	0.67

PIC, picolinic acid; SSRI, selective serotonin reuptake inhibitor; SNRI, serotonin-norepinephrine reuptake inhibitor. There was no effect of any of the groups of medication on the PIC levels except for propiomazine. Patients that received propiomazine had higher levels of PIC in the plasma, therefore we did not correct for this effect in our analysis because it could not be a confounder of the result of reduced PIC in the plasma of patients.
