# Supplementary figures and images for: An enzyme in the kynurenine pathway that governs vulnerability to suicidal behavior by regulating excitotoxicity and neuroinflammation
Source: Transl Psychiatry. 2016 Aug 2;6(8):e865–. doi: 10.1038/tp.2016.133 (PMC5022080; doi:10.1038/tp.2016.133)

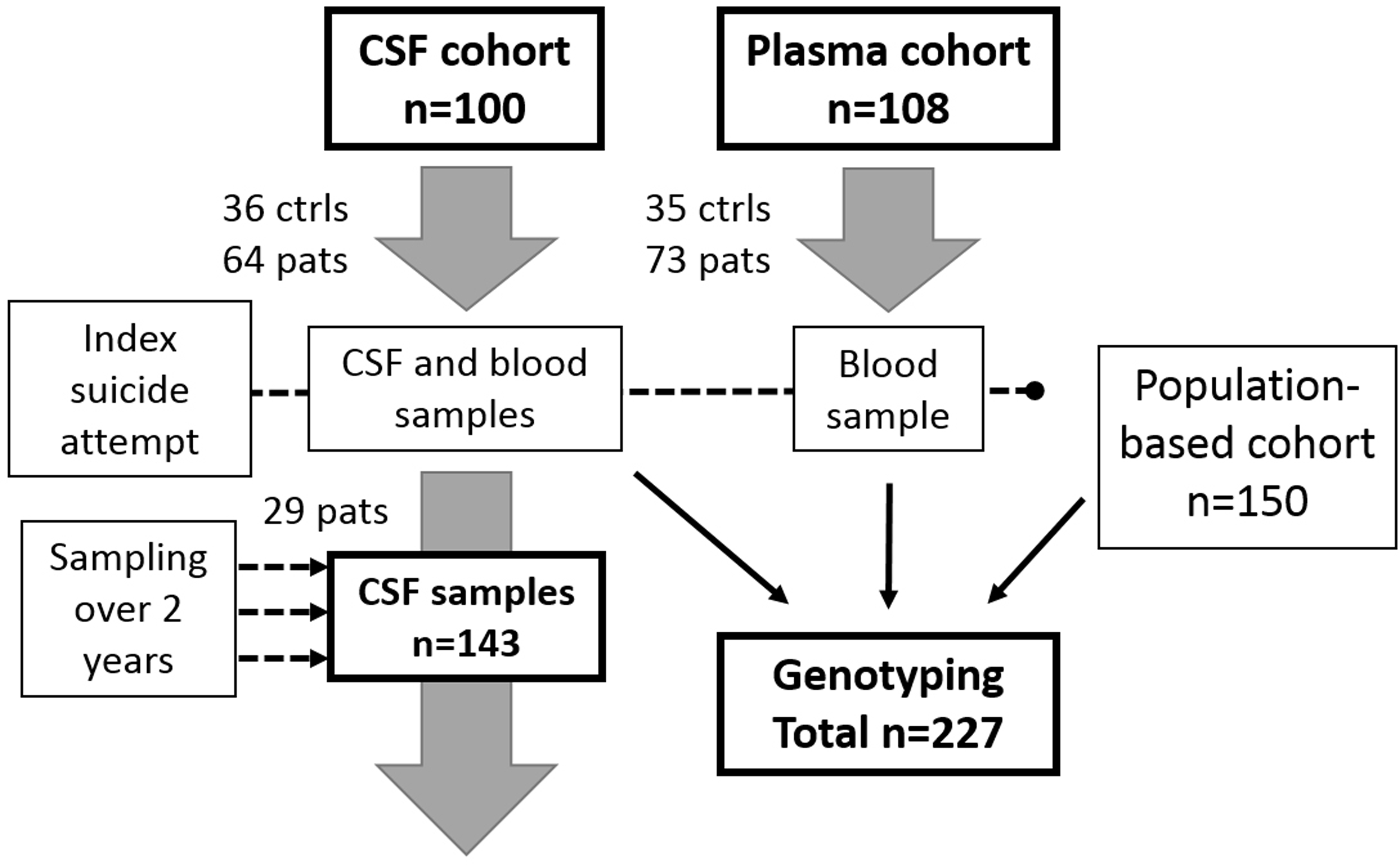

Supplement: Supplementary Figure 1 [file tp2016133x1.tif]

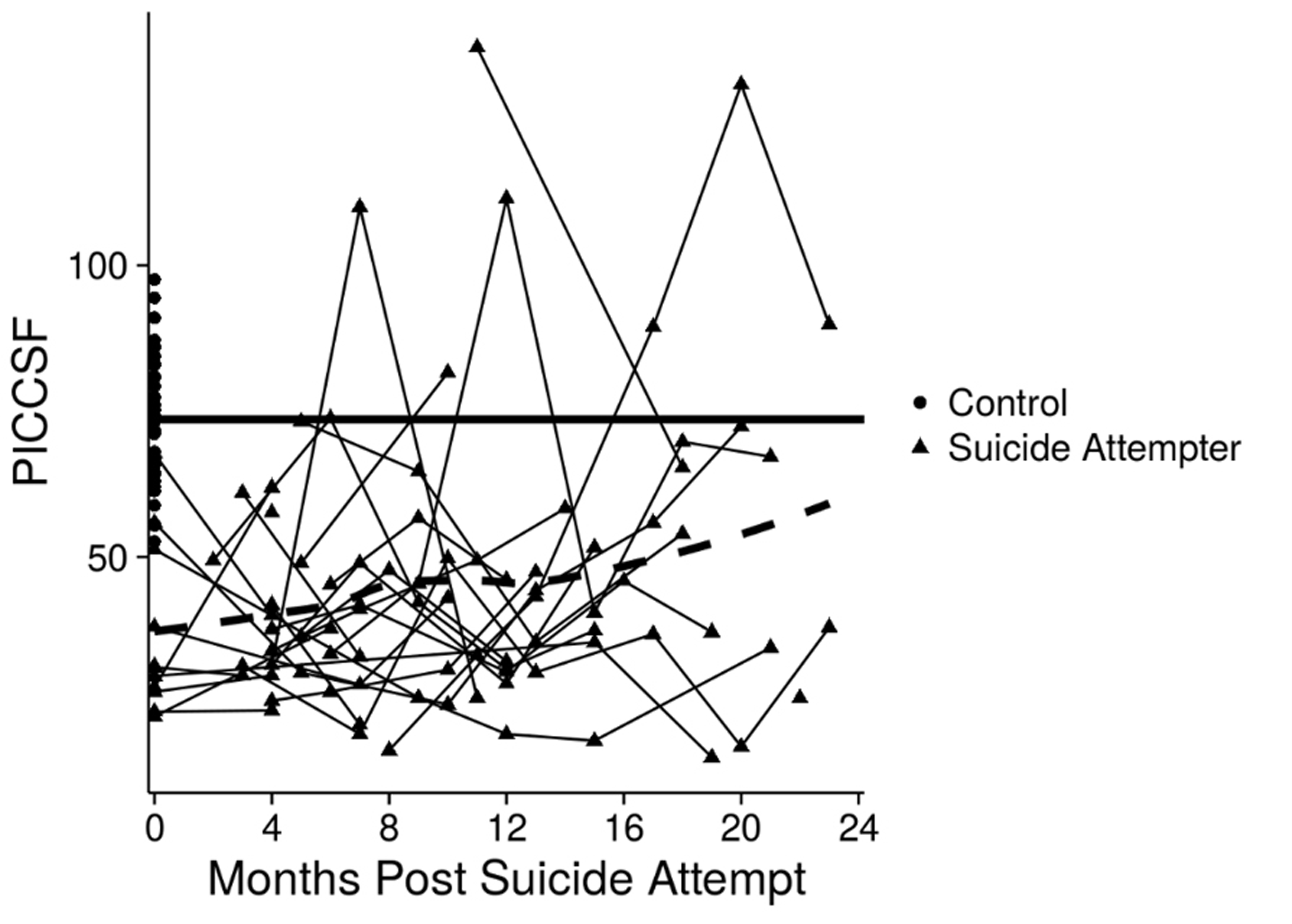

Supplement: Supplementary Figure 2 [file tp2016133x2.tif]
