# Supplementary material for: An enzyme in the kynurenine pathway that governs vulnerability to suicidal behavior by regulating excitotoxicity and neuroinflammation
Source: Transl Psychiatry. 2016 Aug 2;6(8):e865–. doi: 10.1038/tp.2016.133 (PMC5022080; doi:10.1038/tp.2016.133)
Supplement: Supplementary Legends [file tp2016133x4.doc]

**Supplementary Table/Figure legends**

**Supplementary Figure 1**. Schematic of the experimental design. In the CSF cohort, cerebrospinal fluid (CSF) and blood samples were collected from 64 patients who had attempted suicide (pats) and 36 healthy controls (ctrls). 29 of the patients entered the longitudinal study and gave repeated CSF samples for up to 2 years post the index suicide attempt. In the plasma cohort, blood samples were collected from 73 suicide attempters and 35 healthy controls. Whole blood for genotyping was available for 77 suicide attempters from the CSF and plasma cohorts, and a population-based cohort of 150 healthy subjects were genotyped as the control group.

**Supplementary Figure 2.** Picolinic acid (PIC) levels in the cerebrospinal fluid (CSF) of suicide attempters over time. A linear-mixed effects model with random slopes showed that PIC levels did not change significantly over time (Estimate=0.56 nM/month, SEM=0.34, p=0.11), while the delta method showed that patient index scores differed from the control group by an average of 34.82 nM (p=0.0039, SEM=12.08).

**Supplementary Table 1.** Association between the ACMSD_rs2121337_C allele and cerebrospinal fluid (CSF) and plasma analyte levels. Post Bonferroni correction, the minor C allele was associated with increased CSF quinolinic acid (QUIN) levels (β=0.4, p=0.04), and an increased CSF quinolinic acid/picolinic acid (QUIN/PIC) ratio (β=0.4; p=0.02).
